# Enhancing the Mechanical Strength of a Photocurable 3D Printing Material Using Potassium Titanate Additives for Craniofacial Applications

**DOI:** 10.3390/biomimetics9110698

**Published:** 2024-11-14

**Authors:** Yura Choi, Jinyoung Kim, Choongjae Lee, Geonho Lee, Jayoung Hyeon, Soon-ki Jeong, Namchul Cho

**Affiliations:** Department of Energy Engineering, Soonchunhyang University, Asan-si 31538, Republic of Korea; bnbn3238@sch.ac.kr (Y.C.); kjy05007@naver.com (J.K.); dlcndwosla@gmail.com (C.L.); sinb0603@sch.ac.kr (G.L.); jayoung@sch.ac.kr (J.H.)

**Keywords:** 3D printing, craniofacial application, potassium titanate, ceramic additive, photocurable polymer

## Abstract

Photopolymerization-based three-dimensional (3D) printing techniques such as stereolithography (SLA) attract considerable attention owing to their superior resolution, low cost, and relatively high printing speed. However, the lack of studies on improving the mechanical properties of 3D materials highlights the importance of delving deeper into additive manufacturing research. These materials possess considerable potential in the medical field, particularly for applications such as anatomical models, medical devices, and implants. In this study, we investigated the enhancement of mechanical strength in 3D-printed photopolymers through the incorporation of potassium titanate powder (K_2_Ti_8_O_17_), with a particular focus on potential applications in medical devices. The mechanical strength of the photopolymer containing potassium titanate was analyzed by measuring its flexural strength, hardness, and tensile strength. Additionally, poly(ethylene glycol) (PEG) was used as a stabilizer to optimize the dispersion of potassium titanate in the photopolymer. The flexural strengths of the printed specimens were in the range of 15–39 MPa (Megapascals), while the measured surface hardness and tensile strength were in the range of 41–80 HDD (Hardness shore D) and 2.3–15 MPa, respectively. Furthermore, the output resolution was investigated by testing it with a line-patterned structure. The 3D-printing photopolymer without PEG stabilizers produced line patterns with a thickness of 0.3 mm, whereas the 3D-printed resin containing a PEG stabilizer produced line patterns with a thickness of 0.2 mm. These findings demonstrate that the composite materials not only exhibit improved mechanical performance but also allow for high-resolution printing. Furthermore, this composite material was successfully utilized to print implants for pre-surgical inspection. This process ensures the precision and quality of medical device production, emphasizing the material’s practical value in advanced medical applications.

## 1. Introduction

Additive manufacturing (AM), commonly known as three-dimensional (3D) printing, converts computer-aided design (CAD) models into physical objects via layer-by-layer construction [1]. The stereolithography (SLA) 3D-printing technology, based on AM, was invented by Charles Hull in 1986 [2,3]. The SLA 3D-printing technology was introduced to fabricate customized or complex objects without molds or machining [3,4]. Since its inception, 3D printing has proliferated across various industries, particularly in healthcare, where it has emerged as a transformative tool for medical modeling, device fabrication, and presurgical planning. This technology enables the rapid and cost-effective production of anatomical models, prosthetics, and implants customized to meet individual patient needs [5,6,7,8].

Resins used in 3D printing are classified into several types based on their chemical properties and applications [9,10]. Commonly used resins for 3D printing include photopolymers, thermoplastics, epoxy resins, polyurethane resins, and silicone resins. Photopolymers are photocurable, while silicone resins are thermoplastic. Epoxy resins and polyurethane resins are classified as thermosetting. Photopolymers are the most commonly used resins that can be cured via exposure to ultraviolet (UV) light. They are available in various colors, transparencies, and mechanical properties. Thermoplastic resins are suitable for rapid prototyping and lightweight production, whereas epoxy resins provide high strength, durability, and chemical resistance for industrial applications. Polyurethane resins offer flexibility, toughness, impact resistance, and diverse mechanical properties for automotive parts, sports, and medical devices [11,12].

Various photocurable additives with different chemical and mechanical properties have been used in 3D-printing processes. A photoinitiator is required to convert photolytic energy into reactive species that can drive chain growth via radical and cationic mechanisms [13,14,15]. Commonly used photoinitiators include camphorquinone (CQ) and diphenyl (2,4,6-trimethyl benzoyl) phosphine oxide (TPO) [16]. Reactive diluents or stabilizers can be used to control the viscosity of the resin and improve the printing characteristics of 3D printers, whereas UV stabilizers protect against discoloration [17]. The polymers used include poly(ethylene glycol) (PEG), poly(vinyl alcohol) (PVA), and poly(methyl methacrylate) (PMMA). However, excessive use may deteriorate the mechanical properties of the resin. The shrinkage rate during the curing process can be controlled using a reactive diluent, such as 2,2-bis(hydroxymethyl)propane-1,3-diyl trimethacrylate (TMPTMA), 1,2-bis(2-methylacryloyloxy)ethyl ether (TEGDMA), and 3-methacryloyloxypropyltrimethoxysilane. Antioxidants prevent discoloration, brittleness, or cracking of the resin when exposed to oxygen or heat. Reinforcements, such as particles, fibers, and nanomaterials, int the polymers enhance their mechanical properties, exhibiting elevated performance levels and exceptional printing properties [18,19].

The physical and mechanical properties of 3D-printer photopolymers can be reinforced using ceramic-based powders [19,20]. Ceramic-based powders, including zirconia, alumina, and silicon carbide are the most commonly used additives [21,22]. Ceramic resins require a sufficient weight fraction of powder to control the shrinkage, mechanical strength, and density of the resin [23]. Additionally, the effective dispersion of additives forms a uniform layer and decreases defects during structure formation [24]. 

Potassium titanate has been extensively studied owing to its high wear resistance and dielectric properties. Moreover, it has been extensively used in reaction catalysts and environmental purification agents; however, the application of potassium titanate in 3D printing has not been reported thus far [25]. However, potassium titanate offers significant advantages in enhancing mechanical and thermal properties compared to other reinforcements. Studies have shown that the addition of potassium titanate whiskers improves fracture stress and thermal properties in unreinforced ABS copolymers [26]. Furthermore, when incorporated into a nylon-66 matrix, it significantly enhances fracture stress, flexural strength, and impact resistance [27]. Calcium phosphate is a commonly used composite biocompatible polymer in 3D printing for bone tissue repair. However, potassium titanate has also recently gained attention as a promising material in tissue engineering research [28,29,30]. Researchers are focusing on the potential of potassium titanate for use in manufacturing processes that require high-performance materials, as well as its contribution to the development of environmentally friendly and sustainable technologies. Further research in these areas could lead to new innovations and applications across various industries and the medical field [31,32].

In this study, we investigated the enhancement of mechanical strength in 3D-printed photopolymers by incorporating potassium titanate powder (K_2_Ti_8_O_17_). The aim of this research on composite materials is to enable their application in various medical fields, including anatomical models and implants. The mechanical strength, viscosity, flexural strength, tensile strength, and hardness of potassium titanate/acrylate composite were examined at various amounts of potassium titanate additive. Additionally, to address the issue of aggregation of potassium titanate in the photopolymer, PEG was used as a stabilizer to optimize the dispersion of the additive. The results demonstrate the potential of the potassium titanate/acrylate composite for achieving improved mechanical performance and high output resolution in 3D printing applications. Furthermore, prefabricated implants utilizing CAD-based 3D printing can be implemented as prototypes for assessing dimensional accuracy and design suitability. Conducting preliminary tests with these prototypes before using high-cost materials poly(ether ether ketone) (PEEK) and titanium contributes to reducing manufacturing costs and time. Additionally, the preoperative implant models assist in explaining surgical procedures to patients and enable patient-specific customization, which enhances aesthetic and functional outcomes.

## 2. Materials and Methods

### 2.1. Materials

**Figure 1:**
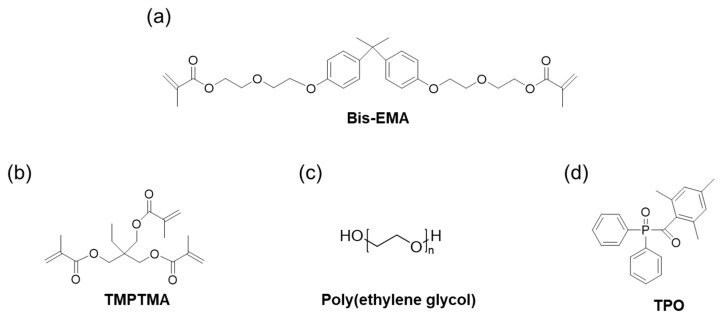
Chemical structures of the materials (**a**) Bis-EMA, (**b**) TMPTMA, (**c**) the stabilizer poly(ethylene glycol) (PEG), and (**d**) the initiator (TPO).

An A+ SLA 3D-printer was used with a UV curing unit (405 nm) (Sindoh Co., Ltd., Seoul, Republic of Korea). Bisphenol-A-ethoxylate dimethacrylate (Bis-EMA) has a molecular structure that imparts both rigidity and flexibility due to the presence of aromatic rings and ethoxylated segments. The trimethylolpropane trimethacrylate (TMPTMA) used in the formulation is recognized for its high cross-linking density, which significantly enhances the mechanical strength of the printed material. Diphenyl (2,4,6-trimethylbenzoyl) phosphine oxide (TPO) functions as a photoinitiator by effectively absorbing UV light at 405 nm and initiating the polymerization process. It was purchased from MIWON Chemical Co., Ltd. (Yongin-si, Republic of Korea). The bi-functional aliphatic urethane acrylate oligomer obtained from Asan Materials Co., Ltd. (Asan-si, Republic of Korea) enhances the toughness and flexibility of the printed structures. Its aliphatic backbone provides UV stability, thus ensuring long-term durability under UV exposure. A poly(ethylene glycol) (PEG 300) is used as a stabilizer and is sourced from Tokyo Chemical Industry Co., Ltd. (Tokyo, Japan). It improves the dispersion of components and enhances the printability of the resin by reducing its viscosity. Potassium titanate (K_2_Ti_8_O_17_) was purchased from New Materials Co., Ltd. (Asan-si, Republic of Korea) (Figure 1). This can improve mechanical properties with high durability and wear resistance. The yellow 83 pigment was purchased from Heubach Color Pvt. Ltd. (Ankleshwar, India).

### 2.2. Method

The compositions of the various 3D-printing resin composites prepared are listed in Table 1. The photopolymer bases used in the resin composites were synthesized with a ratio of Bis-EMA:TMPTMA:oligomers = 7.5:1:0.5 wt%. The photoinitiator amount was 4 wt% based on the total weight of the photopolymer base [33]. The photopolymer bases were prepared via ball milling at 150 rpm for 10 h (DAIHAN Scientific, Daegu, Republic of Korea, BML-2). The base resin was allowed to sit at room temperature to remove air bubbles before use. The PEG additive and potassium titanate powder were dispersed in the photopolymer base via ball milling at 150 rpm for 8 h (DAIHAN Scientific, Daegu, Republic of Korea, BML-2).

The additives were used to prepare PEG/potassium titanate/acrylate composites using the same ratios as in Table 1. The PEG additive and potassium titanate powder were dispersed in the photopolymer base via ball milling at 150 rpm for 8 h (DAIHAN Scientific, Daegu, Republic of Korea, BML-2). The additive was manufactured using the same weight ratio as in Table 1.

#### 2.2.1. Analysis of Viscosity

The viscosities of the photopolymers were determined using a viscometer (DV2TLV Rheometer, Brookfield, Toronto, ON, Canada). The temperatures of the specimens were maintained in the range of 22–25 °C. Readings were recorded at 10 rpm for 5 min in a centipoise. Each photopolymer was analyzed five times, and the average value was calculated. 

#### 2.2.2. Analysis of Flexural Strength

The flexural strength was determined through a 3-point bending test. Rectangular specimens (1 cm × 8 cm × 0.5 cm) were prepared using a 3D printer. The printed specimen was initially cleaned on the surface with isopropyl alcohol (IPA). Subsequently, it was immersed in IPA and subjected to an ultrasonic cleaner for 1 to 2 min to remove any residues. After the cleaning process, the surface was subjected to a photocuring process. The photocuring process was performed using a UV Curing Machine with a power of 80 W and a wavelength of 405 nm. The curing time for the samples was approximately 5 min (3D UV Curing Machine 80 W, Ningbo, China).

Flexural strength tests were performed using a universal testing machine (UTM; AGS-X, Shimadzu Corporation, Seoul, Republic of Korea) (Figure S1a). The specimens were immersed in a distilled water bath (37 ± 1 °C) for 25 h, and their thicknesses and widths were measured. The specimens were measured using the 3-point jig of a UTM (measurement speed: Max 1000 mm/min). Flexural strength testing involved the fabrication of 11 specimens for each resin material, and measurements were performed in triplicate for each specimen to calculate the average values (Figure S2a).

#### 2.2.3. Analysis of Hardness

Various ratios of monomer photopolymers were used to analyze the hardness of the specimens. Specimens measuring 2 cm × 2 cm × 2 cm were prepared using a 3D printer. The printed specimens were cleaned in the same manner as those used for the flexural strength test. Subsequently, the specimens were cured using a UV curing machine with a power of 80 W and a wavelength of 405 nm for approximately 5 min to polymerize any unreacted monomers. The hardness was measured using a Durometer Digital Shore D system (Zwcik 3131, ZwickRoell GmbH & Co. KG, Ulm, Germany) (Figure S1b). Each measurement was conducted for 0–99 s with a force of 44.5 N at a truncated cone opening angle of 30°. Eleven specimens per resin material were produced, and the measurements were conducted in triplicate per specimen to obtain the average values (Figure S2b).

#### 2.2.4. Analysis of Tensile Strength

The specimens for the tensile strength measurements were prepared according to the specifications (full-length L_3_ = 150 mm, parallel length L_2_ = 60.0 ± 5 mm, gauge length L_1_ = 108 ± 1.6 mm, thickness T = 4.0 ± 2 mm) using a 3D printer. The 3D-printed specimens were post-processed following the same procedure as with the hardness measurements. The tensile strength was measured using a Zwick/Roell (Z150) tester (ZwickRoell GmbH& Co. KG, Ulm, Germany) at a cross-head speed of 1 mm/min. Eleven specimens were prepared for each photopolymer material, and the measurements were conducted in triplicate per specimen to calculate the average value.

#### 2.2.5. Preparing the 3D Printer for Output

Line patterns were designed using HAN CAD, and line pattern support was also prepared (width = 3.2 cm; length = 6 cm; height = 3 mm). The line thickness range of the entire line pattern was in the range of 0.2–5 mm. The line pattern thickness was divided into ranges of 0.2–1 mm and 1–5 mm. Line patterns were designed with a thickness of 9 lines, ranging from 0.2 to 1 mm in increments of 0.1 mm. Additionally, a 5-line thickness pattern was created, spanning from 1 to 5 mm in increments of 1 mm. The craniofacial implant was redesigned in CAD using CT and other imaging files provided by the hospital. Both the line patterns and the implant were printed with an A+ SLA 3D printer, which utilizes a 405 nm/Laser diode. The laser output was set at 450 mW (Figure S1c,d).

## 3. Results and Discussion

In this study, photopolymers were prepared by mixing acrylate monomers (Bis-EMA), a bi-functional aliphatic urethane acrylate oligomer, and a crosslinker (TMPTMA) (Figure 1). Potassium titanate was used as a ceramic additive to improve the mechanical strength of the resin. Potassium titanate was added at 0.5 wt% increments to achieve a gradual variation in viscosity [34]. A high weight fraction of additives is important for improving the mechanical strength of the photopolymer. However, addressing the issue of agglomeration of ceramic photopolymers is also essential when a large amount of ceramic photopolymer is used. If the dispersibility of the ceramic photopolymer is low, defects will occur in the final printed structure. Thus, PEG was used as a stabilizer to prevent the aggregation of potassium titanate in the photopolymer. PEG is a hydrophilic polymer comprising -OH end groups, able to reduce interactions between ceramic additive particles, preventing agglomeration and precipitation [35]. Generally, the maximum amount of PEG in a photocurable photopolymer is approximately 10 wt%; amounts higher than 10 wt% can decrease the mechanical strength of the photocrosslinked polymers [24]. 

**Figure 2:**
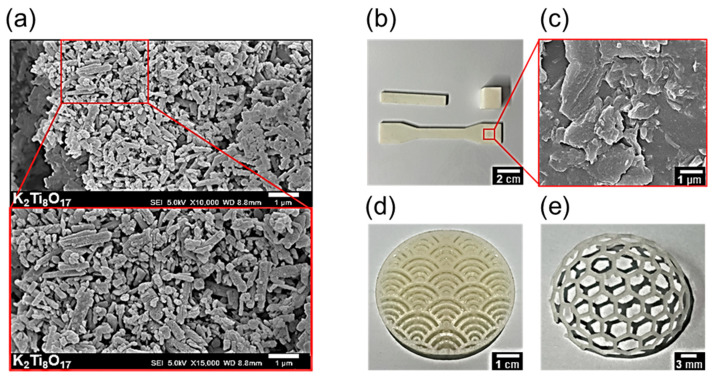
(**a**) Scanning electron microscopy (SEM) image of the potassium titanate powder (10,000× magnification and 15,000× magnification), (**b**) printed specimens (PT15) for mechanical strength measurements, and (**c**) their SEM image. (**d**) Digital photos of the 3D-printed curved pattern structure and (**e**) hexagonal hemisphere.

Figure 2a shows the SEM images of potassium titanate powder. Potassium titanate powder was prepared by ball milling at 120 rpm for 24 h. After ball milling, the powder was separated using a 45 μm sieve. The diameters of the potassium titanate particles were in the range of 0.09–1.13 μm, with an average diameter of approximately 0.45 μm. The resolution of the acrylate photopolymer used for SLA can vary depending on the depth of light penetration. Figure 2c shows the SEM image of the specimen prepared for the mechanical strength teste. The curved pattern structure in Figure 2d was printed using a potassium titanate/acrylate composite (potassium titanate:acrylate photopolymer base:PEG = 1:89:10 wt%). Figure 2e shows the hemisphere structure printed using the potassium titanate/acrylate composite (potassium titanate:acrylate photopolymer base:PEG = 0.5:89.5:10 wt%). The potassium titanate/acrylate composite successfully produced structures without any defects or delamination, which is critical for 3D printer applications.

In this study, a photoinitiator with an absorption peak in the range of 350–420 nm was used for the photopolymerization reaction. Typically, CQ and TPO are used as initiators for the photopolymerization of resins. CQ has a visible-light absorption peak in the range of 380–520 nm, while TPO exhibits an absorption peak in the range of 380–425 nm. In this study, TPO was used as the initiator for the potassium titanate/acrylate composite owing to its higher polymerization efficiency compared to CQ. In addition, the TPO initiator changes from a pale yellow color to transparent upon exposure to UV light. 

### 3.1. Viscosity of Potassium Titanate/Acrylate Composite

**Figure 3:**
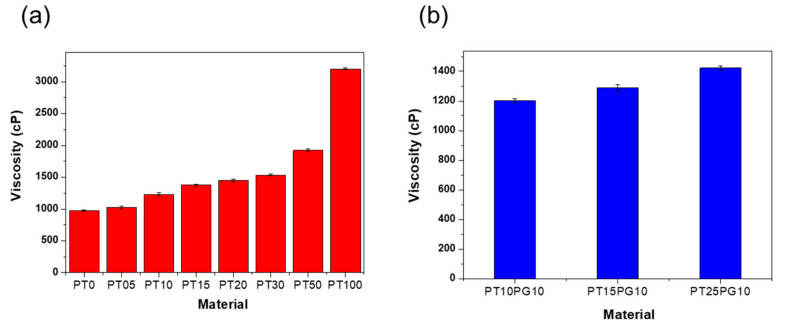
Viscosities of the 3D-printed composites. (**a**) Potassium titanate/acrylate composite and (**b**) PEG/potassium titanate/acrylate composite.

The number of acrylate and methacrylate groups in photocurable monomers generally affects the crosslinking reaction and the mechanical properties of the products. Furthermore, the photopolymer viscosity, co-monomer concentration, monomer structure, and photoinitiator affect the polymerization rate and resolution [36,37,38]. SLA 3D printing requires the use of uncured photopolymers with low viscosities for the successful coating of the base layer as well as the main body [39]. Especially for photopolymers containing ceramic powders, their viscosity should be less than 3000 cP for the self-leveling step during the 3D printing process [40,41]. As the concentration of ceramic powder increases, the number of solid particles dispersed within the resin also increases. These particles introduce additional resistance to the flow of the resin, resulting in an increase in viscosity. As shown in Table 2 and Figure 3, the viscosity increases significantly with the weight fraction of the additive. The viscosity of the potassium titanate/acrylate composite was in the range of 3202-975 cP, and the amount of additive used was limited to a maximum of 10 wt% [20,42]. 

The viscosity of PT0 and PT100 was 975 and 3202 cP, respectively, exhibiting a difference of 3.2 times. For photopolymers with a viscosity of 3000 cP or higher, misprinting and undispersed potassium titanate powder was observed in the printed structure. When the viscosity of the potassium titanate/acrylate composite exceeded 2000 cP, missing layers appeared in the resin printouts. Above PT15, a small amount of PEG additive was added to control the viscosity and dispersibility of the photopolymer (Table 1). PT15 exhibited a viscosity of 1375 cP, whereas that of PT15PG10, containing the PEG additive, decreased to 1289 cP (Figure 3b). 

### 3.2. Mechanical Properties of Potassium Titanate/Acrylate Composite

The number of acrylate and methacrylate groups in photocurable monomers generally affects the crosslinking reaction and the mechanical properties of the products. 

Generally, increasing the weight fraction of the ceramic additives enhances the mechanical strength of the resin. The maximum weight fraction of additives depends on the type of monomer, stabilizer, and diluent used. However, a higher additive content may lead to increased viscosity and decreased light transmittance and resolution. Additionally, exceeding the maximum weight fraction can hinder dispersion, leading to aggregation and a decrease in mechanical strength [43,44]. 

**Figure 4:**
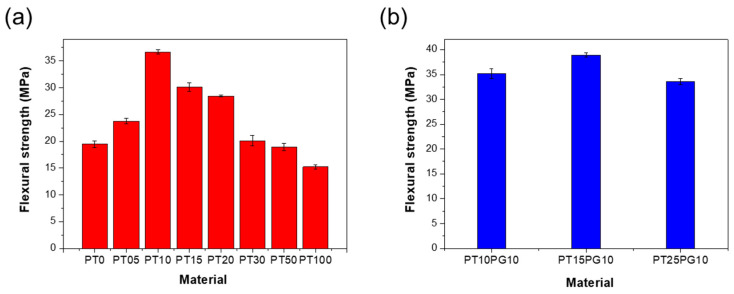
Flexural strength of 3D-printed specimens. (**a**) Potassium titanate/acrylate composite and (**b**) PEG/potassium titanate/acrylate composite.

As summarized in Table 3, the mechanical properties of the potassium titanate/acrylate composite first increase and then decrease with an increase in the potassium titanate powder content. PEG was used as a stabilizer to inhibit the aggregation of potassium titanate. The flexural strength values are shown in Figure 4. PT10 exhibited the highest flexural strength of 36.63 ± 0.4 MPa, whereas PT100 exhibited the lowest flexural strength of 15.21 ± 0.4 MPa. Figure 4a shows that the flexural strength was the highest when the potassium titanate content was 1 wt%; a potassium titanate content higher than 1 wt% caused a decrease in flexural strength. However, compared to PT15, PT15PG10 (containing PEG) exhibited a higher flexural strength (Figure 4b), with its highest flexural strength reaching 38.93 ± 0.4 MPa [45].

**Figure 5:**
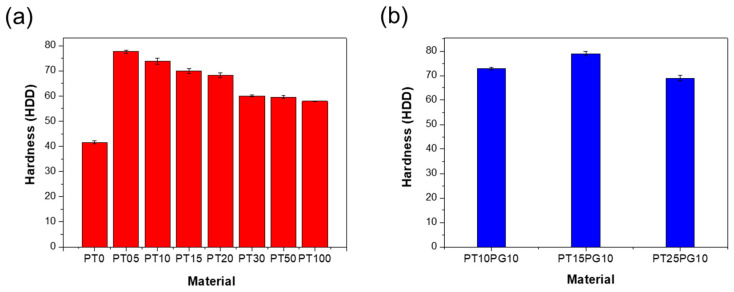
Surface hardness of the 3D-printed specimens. (**a**) Potassium titanate/acrylate composite and (**b**) PEG/potassium titanate/acrylate composite.

Figure 5 shows the surface hardness values using a Zwick 3131 indenter (θ = 30°). The indenter is pressed into the specimen with a spring force ranging from 1.11 to 44.5 N. Subsequently, the indenter is pressed onto the specimen with a contact force varying from 3.92 to 50 N for a duration of 0 to 99 s. The indentation depth of the specimens ranged from 0 to 2.5 mm. The penetration depth is measured using a hardness tester, from which the hardness value is determined. 

The PT15PG10 3D-printed cube exhibited the highest surface hardness of 79.0 ± 0.9 HDD. PT0 showed the lowest surface hardness values of 41.5 ± 0.6 HDD. The surface hardness of the resin containing a high amount of potassium titanate was lower than that of the resin containing 10 wt% PEG. Even with the same amount of potassium titanate, the surface hardness of the resin containing PEG is higher (Figure 5b). The surface hardnesses of PT15 and PT15PG10 were 70.0 ± 1.0 and 79.0 ± 0.9 HDD, respectively, exhibiting a difference of approximately 11%. PT15PG10, containing PEG, exhibited the highest values, indicating that the PEG stabilizer promoted the even dispersion of the potassium titanate powder in the photopolymer (Figure S3). Potassium titanate is evenly distributed throughout the resin, thus increasing its strength by reducing stress concentration and preventing misprinting and pore formation.

**Figure 6:**
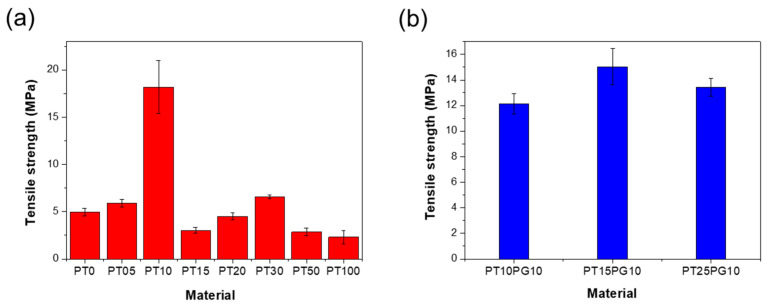
Tensile strength of the 3D-printed specimens. (**a**) Potassium titanate/acrylate composite and (**b**) PEG/potassium titanate/acrylate composite.

The tensile strength curve in Figure 6 demonstrates a somewhat different trend when compared to the flexural strength results. Specifically, the PT10 specimen exhibited the highest tensile strength at 18.2 ± 2.8 MPa. However, as the potassium titanate content increased beyond 1 wt%, the tensile strengths generally decreased, with the PT100 specimen showing the lowest tensile strength at 2.32 ± 0.7 MPa. The observed increase in tensile strength from PT15 to PT30 can be attributed to improved particle dispersion within the resin at these concentrations. Enhanced dispersion results in a more uniform distribution of potassium titanate particles, which more effectively reinforces the polymer matrix under tensile stress. This reinforcement effect is particularly pronounced during tensile testing, as the stress is distributed more evenly across the material, enabling the dispersed particles to bear the load more effectively. The differences in behavior observed between tensile and bending tests reflect a complex interaction between particle dispersion and the specific type of mechanical stress applied during testing. These factors can lead to varying results in material strength, depending on the test method used. The differences in tensile strength among the samples without PEG exceeded 10 MPa, with the exception of the PT10 specimen. This indicates that while PEG aided in dispersion and enhanced tensile strength, the specific content and distribution of potassium titanate remained crucial to the overall mechanical properties.

Young’s modulus also exhibited a similar trend to the tensile strength (Figure S4). For potassium titanate amounts exceeding 1 wt%, Young’s modulus decreased. However, the specimens containing PEG showed a tendency to increase in proportion to the potassium titanate content. The PT25PG10 specimen exhibited the highest Young’s modulus of 386 MPa (Table S1). 

Photopolymerized composites have interfaces between the different soft and stiff phases that are covalently bonded after radical polymerization using ultraviolet light. Covalently bonded composites exhibit enhanced flexural and tensile strengths [46]. Initial destruction of the composite resin occurs during the additive/matrix interphase. The rigidity of the material contributes to the distribution of stresses that occur when loaded. Differences in the amount and size of additives alter the surface-area-to-volume ratio, thereby affecting the surface energy of the interphase. Therefore, the stress concentration in the additive/matrix interphase depends on the particle size and additive content, which can consequently increase the flexural strength values of the composite [47]. However, in this study, the strength of the composite first increased and then decreased as the potassium titanate content increased. A high sedimentation rate was observed when the powder additive content exceeded 10%. As the degree of conversion of the crosslinking monomers increased, the density of the polymer network also increased, resulting in enhanced mechanical properties. However, the PT50 composite showed significantly lower mechanical properties because of the high loading of additives. This can be attributed to the undispersed particles within the resin, which tend to obstruct or reflect the laser light during photopolymerization. 

In certain samples (PT50 and PT100), additive particles were observed that were not uniformly distributed on the surfaces of the printed structures. Undispersed potassium titanate causes misprinting and pore formation in the printed structure, resulting in reduced mechanical strengths. Undispersed particles tend to move around within the photopolymer, blocking or reflecting laser light. Alternatively, they may precipitate in a 3D printer bath and suppress laser irradiation. Misprinting and pores occurred in the areas that were not exposed to light, signifying that the addition of potassium titanate powder resulted in a corresponding increase in the amount of residual photopolymer content. According to previous studies, the unpolymerized residual photopolymer reduces the mechanical strength of the material [1,48]. 

The viscosity of the photopolymer decreases by adding a PEG stabilizer and suppresses the interference from any undispersed potassium titanate. The flexural strength of PT15 was 30.12 ± 0.8 MPa. However, the flexural strength of PT15PG10 containing the PEG stabilizer was 38.93 ± 0.4 MPa. The hardness of PT15 was 70.0 ± 1.0 MPa, and that of PT15PG10 was 79.0 ± 0.9 MPa. Therefore, the incorporation of potassium titanate increases the mechanical strength of the photopolymer and aids in preventing the occurrence of misprints and pores within the photopolymer structure by acting as a physical barrier. Despite the irregular and non-uniform characteristics of the particles, their presence can still obstruct crack propagation paths and enhance the material’s overall toughness [49].

### 3.3. Three-Dimensional Printing of Potassium Titanate/Acrylate Composite

**Figure 7:**
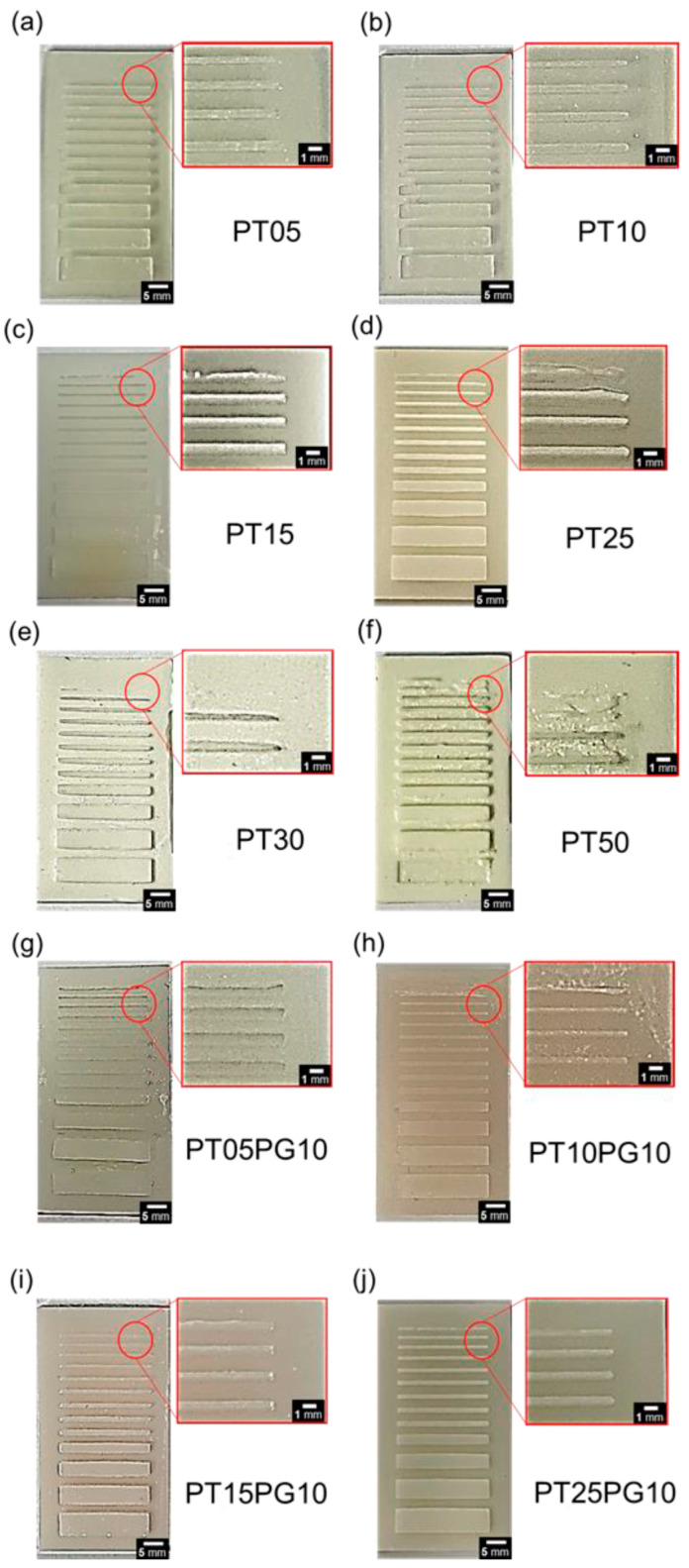
Three-dimensionally printed line pattern using the potassium titanate/acrylate composites (the line thickness ranges from 0.2 to 5 mm) as a photoinitiating system for the polymerization of the 3D resin. Line pattern images printed with a resin containing (**a**) 0.5 wt% of potassium titanate, (**b**) 1.0 wt% of potassium titanate, (**c**) 1.5 wt% of potassium titanate, (**d**) 2.5 wt% of potassium titanate, (**e**) 3.0 wt% of potassium titanate, (**f**) 5.0 wt% of potassium titanate, (**g**) 0.5 wt% of potassium titanate and 10 wt% PEG, (**h**) 1.0 wt% of potassium titanate and 10 wt% PEG, (**i**) 1.5 wt% of potassium titanate and 10 wt% PEG, and (**j**) 2.5 wt% of potassium titanate and 10 wt% PEG.

The strengths of the SLA printers lie in their excellent resolution and precision. Therefore, a resolution test is necessary for validating the use of potassium titanate as an additive in photopolymers. Generally, ceramic additives can interfere with the photopolymerization process or reduce light penetration owing to light scattering, depending on their concentration. Light scattering is associated with photopolymer turbidity. The turbidity of the photopolymer is significantly affected by the concentration of ceramic additives. Higher turbidity may result in less absorption or scattering of light [50,51]. Generally, photopolymers for 3D printers available on the market exhibit a minimum resolution of 0.2–0.3 mm. As shown in Figure 7, the resin with the added PEG stabilizer is printed using an SLA 3D printer. Under identical printing conditions (fixed scan speed), the potassium titanate photopolymer composite with an added PEG stabilizer demonstrated excellent accuracy. As shown in Figure 7, the resin with a lower amount of potassium titanate exhibited a better line pattern resolution. In addition, potassium titanate photopolymer composites with added PEG stabilizers achieved a minimum line pattern length of 0.2 mm.

As shown in Figure 7, PT05, PT10, PT05PG10, PT10PG10, PT15PG10, and PT25PG10 were printed without misprinting. Furthermore, PT15 and PT25 printed line patterns of up to 0.3 mm. In the case of PT30, potassium titanate particles were present on the surface of the structure. Figure S5 shows a surface image of a specimen printed using the potassium titanate/acrylate composite. In addition, misprinting appeared throughout the PT50 structure, indicating that the photopolymerization of the resin is suppressed owing to excessive additives. Compared with the CAD file, the error (minimum length of the line pattern) of the PEG stabilizer-added potassium titanate photopolymer composite was 0.05%. Consequently, a resin printed with a very thin and detailed pattern exhibits good resolution as a photopolymer for 3D printers. 

**Figure 8:**
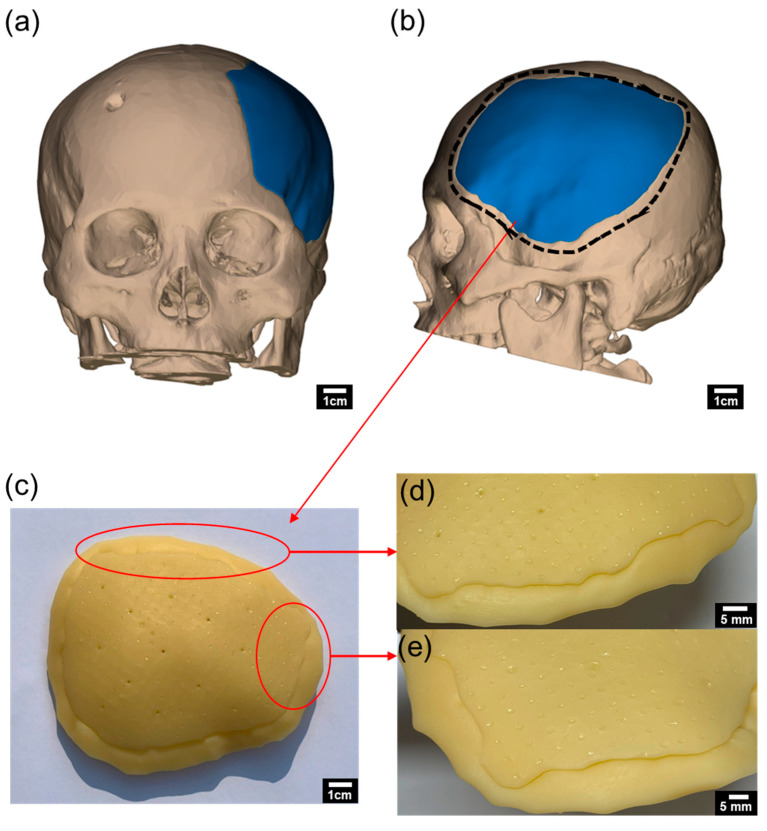

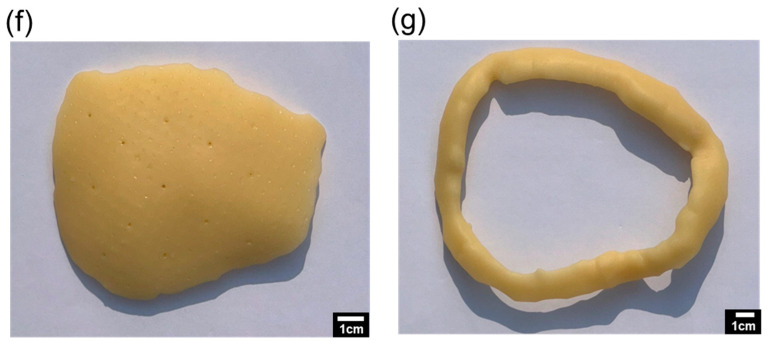
CAD-designed craniofacial implant: (**a**) anterior view, (**b**) lateral view of the implant design, (**c**) 3D-printed craniofacial implant using a potassium titanate/acrylate composite, (**d**,**e**) close-up views of the implant interface for attachment, (**f**) craniofacial implant main body, (**g**) and skull interface area for implant attachment.

The physical and mechanical properties of potassium titanate/acrylate composites make them suitable for a wide range of applications. Figure 8 illustrates a craniofacial implant model used in orthopedics, created by incorporating 0.3 wt% yellow pigment into PT25PG10. Figure 8a,b show the design generated using a CAD program based on CT scan imaging data. The blue region indicates the area where the implant will be attached. After the entire skull was designed, 3D printing was used to fabricate up to the interface boundary for implant attachment (indicated by the black dashed line). As shown in Figure 8c, the craniofacial implant and the skull interface section were printed. Precise alignment at the contact surface between the skull and implant is required to avoid lifting or misalignment. Figure 8d,e demonstrate that the interfaces match accurately, without any lifting or alignment errors. 

Prefabrication using 3D printing enhances product quality and allows for more detailed explanations of the surgical procedure for patients. CAD-based production also enables patient-specific design and flexible modifications. The craniofacial implant in Figure 8 serves as a temporary model. After final inspection and adjustments, the implant is produced in its final form using materials such as titanium and PEEK. Such prefabricated implants can function as prototypes for verifying the dimensions and design of CAD models. Additionally, conducting preliminary tests before utilizing costly materials like PEEK and titanium can significantly reduce manufacturing costs and time. The preoperative implant model further assists in illustrating the surgical procedure to patients and offers a tailored design, thereby enhancing aesthetic outcomes.

The biocompatibility of potassium titanate makes it suitable for various applications in tissue engineering. When immersed in simulated body fluids, potassium titanate demonstrates structural stability and promotes hydroxyapatite formation, essential for bone integration. This biologically active surface supports osteoblast adhesion and proliferation, making it an ideal reinforcing material for bone regeneration implants. Additionally, the ion-exchange properties of potassium titanate facilitate the formation of a calcium titanate layer, enhancing compatibility with bone tissue [29,30,52,53]. 

Further optimization is required to apply potassium titanate/acrylate composites in medical fields. Specifically, additional research is needed to evaluate their durability, thermal stability, toxicity, and biocompatibility for medical applications.

## 4. Conclusions

In this study, potassium titanate powder was used as an additive to enhance the mechanical strength of acrylate photopolymers. The mechanical strength and resolsution of the resin were compared before and after using the PEG stabilizer. The addition of a PEG stabilizer lowered the viscosity of the photopolymer and suppressed the interference from any undispersed potassium titanate. The PEG stabilizer enhanced the bonding forces with the photopolymer matrix, resulting in the increased mechanical strength of the photopolymer. A line pattern with a thickness of 0.2 mm was successfully printed using PEG, potassium titanate, and the acrylate photopolymer. Furthermore, models featuring complex structures were successfully printed, further demonstrating the applicability of the potassium titanate/acrylate composite. Our study reveals valuable insights into the properties of 3D-printing materials, offering a valuable tool for further research on ceramic resins, which is necessary for developing a 3D printer market and advancing 3D-printing technology. Following this study, our team will proceed with the commercialization of potassium titanate and the acrylate photopolymer while examining the stability, biocompatibility, and thermal stability of potassium titanate-based resins.

## Figures and Tables

**Table 1:** Resin compositions of the PEG/potassium titanate/acrylate composites.

Name	Composition (wt%)		
	Photopolymer Base	PEG	Potassium Titanate
PT0	100	0	0
PT05	99.5	0	0.5
PT10	99	0	1.0
PT15	98.5	0	1.5
PT20	98	0	2.0
PT30	97	0	3.0
PT50	95	0	5.0
PT100	90	0	10.0
PT10PG10	89	10	1.0
PT15PG10	88.5	10	1.5
PT25PG10	87.5	10	2.5

**Table 2:** Viscosity values of the various PEG/potassium titanate/acrylate composites.

Name	Viscosity (cP)
PT0	975 ± 10.0
PT05	1025 ± 19.1
PT10	1231 ± 22.0
PT15	1375 ± 15.8
PT20	1452 ± 21.4
PT30	1532 ± 14.6
PT50	1922 ± 19.5
PT100	3202 ± 17.4
PT10PG10	1201 ± 12.7
PT15PG10	1289 ± 21.3
PT25PG10	1422 ± 15.4

**Table 3:** Mechanical strengths of the different ball-milled PEG/potassium titanate/acrylate composite dispersions.

Name	Flexural Strength (MPa)	Hardness (HDD)	Tensile Strength (MPa)
PT0	19.45 ± 0.6	41.5 ± 0.6	4.95 ± 0.4
PT05	23.74 ± 0.5	77.7 ± 0.6	5.93 ± 0.4
PT10	36.63 ± 0.4	73.9 ± 1.2	18.2 ± 2.8
PT15	30.12 ± 0.8	70.0 ± 1.0	3.03 ± 0.3
PT20	28.45 ± 0.2	68.2 ± 0.9	4.51 ± 0.4
PT30	20.11 ± 1.0	60.0 ± 0.4	6.56 ± 0.2
PT50	18.94 ± 0.7	59.5 ± 0.6	2.86 ± 0.4
PT100	15.21± 0.4	57.9 ± 0.2	2.32 ± 0.7
PT10PG10	35.18 ± 1.0	73.0 ± 0.5	12.14 ± 0.8
PT15PG10	38.93 ± 0.4	79.0 ± 0.9	15.04 ± 1.4
PT25PG10	33.54 ± 0.6	69.0 ± 1.1	13.42 ± 0.7

## Data Availability

Data are contained within the article and Supplementary Materials.

## Supplementary Materials

The following supporting information can be downloaded at https://www.mdpi.com/article/10.3390/biomimetics9110698/s1, Figure S1. Equipment used for mechanical strength testing and 3D printing processes. Figure S2. Mechanical strengths of the different specimens. Figure S3. Precipitation amounts of potassium titanate based on resins. Figure S4. Young’s modulus of 3D-printed specimens. Table S1. Young’s modulus values of the various PEG/potassium titanate/acrylate composites. Figure S5. SEM image of the specimen surface containing potassium titanate/acrylate composites.
