# Supplementary material for: Enhancing the Mechanical Strength of a Photocurable 3D Printing Material Using Potassium Titanate Additives for Craniofacial Applications
Source: Biomimetics (Basel). 2024 Nov 14;9(11):698. doi: 10.3390/biomimetics9110698 (PMC11592189; doi:10.3390/biomimetics9110698)
Supplement: Supplementary file 1 [file biomimetics-09-00698-s001.zip › biomimetics-3297815-supplementary.pdf]

biomimetics

Supplementary Materials

Enhancing the Mechanical Strength of a Photocurable 3D Printing Material Using Potassium Titanate Additives for Craniofacial Applications

Yura Choi, Jinyoung Kim, Choongjae Lee, Geonho Lee, Jayoung Hyeon, Soon-ki Jeong * and Namchul Cho *

Department of Energy Engineering, Soonchunhyang University, Asan, 31538, Republic of Korea; bnbn3238@sch.ac.kr (Y.C.); kjy05007@naver.com (J.K.); dlcndwosla@gmail.com (C.L.); sinb0603@sch.ac.kr (G.L.); jayoung@sch.ac.kr (J.H.)
* Correspondence: hamin611@sch.ac.kr (S.-k.J.); chon7@sch.ac.kr (N.C.)

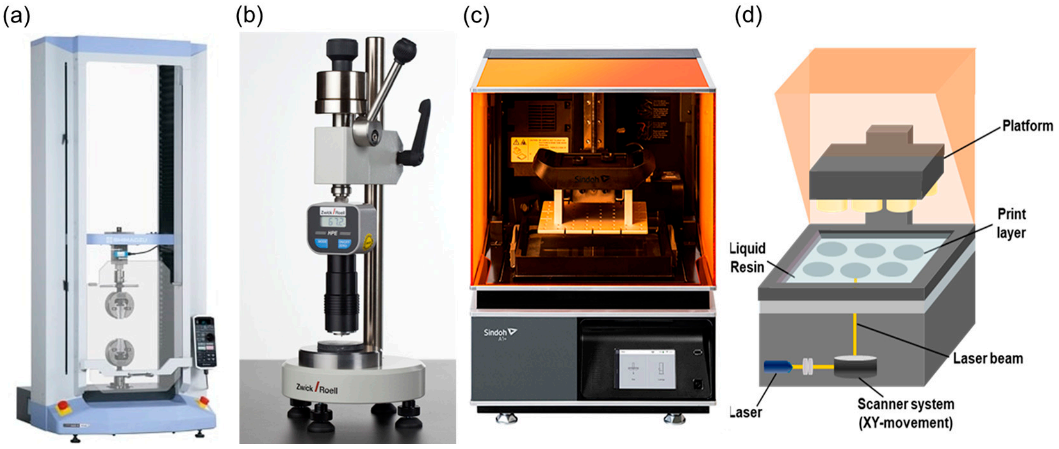

Figure S1. Equipment used for mechanical strength testing and 3D printing processes: (a) flexural strength (UTM; AGS-X), (b) surface hardness (Zwcik 3131), (c) SLA 3D Printer (A+), (d) schematic of the SLA setup.

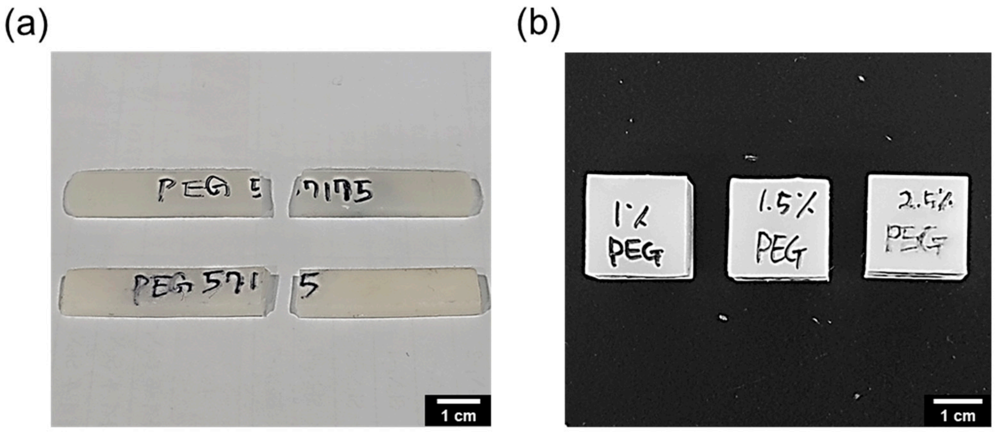

Figure S2. Mechanical strengths of the different specimens. (a) Flexural strength of the specimen containing potassium titanate/acrylate composite. (b) Hardness of the specimen containing potassium titanate/acrylate composite.

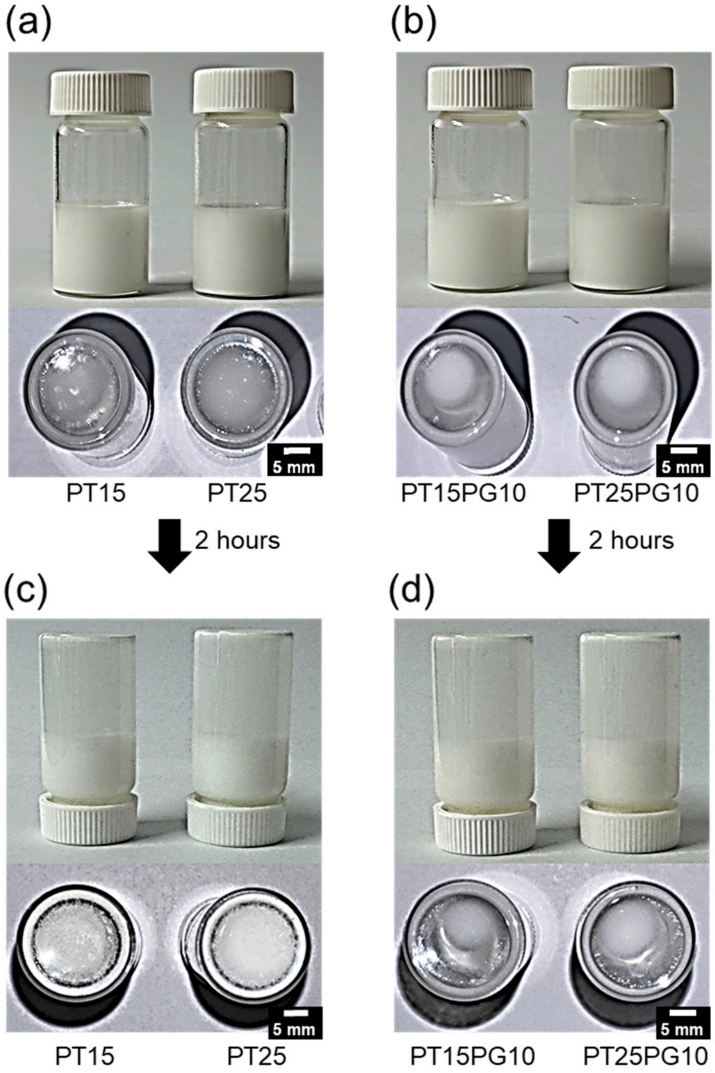

Figure S3. Precipitation amounts of potassium titanate based on resins. (a) Resins mixed with potassium titanate, (b) resins mixed with potassium titanate and PEG, (c) a precipitate formed due to the undispersed of potassium titanate particles, and (d) a resin containing fewer undispersed particles due to the inclusion of PEG.

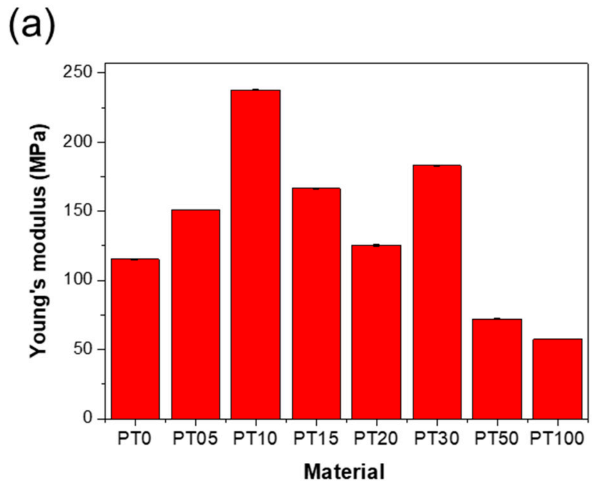

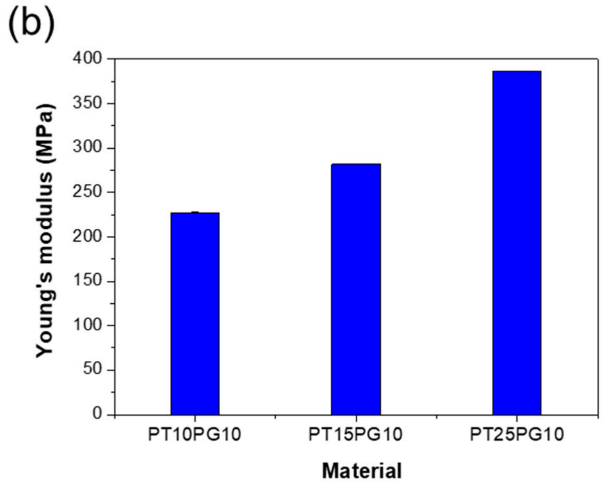

Figure S4. Young's modulus of 3D-printed specimens. (a) Potassium titanate/acrylate composite and (b) PEG/potassium titanate/acrylate composite.

Table S1. Young's modulus values of the various PEG/potassium titanate/acrylate composites.

Name	Young's modulus (MPa)
PT0	115.42 ± 0.5
PT05	151.06 ± 0.3
PT10	237.94 ± 0.7
PT15	166.44 ± 0.2
PT20	125.44 ± 0.5
PT30	183.09 ± 0.6
PT50	72.13 ± 0.3
PT100	57.5 ± 0.3
PT10PG10	227.31 ± 0.5
PT15PG10	281.61 ± 0.3
PT25PG10	386.1 ± 0.4

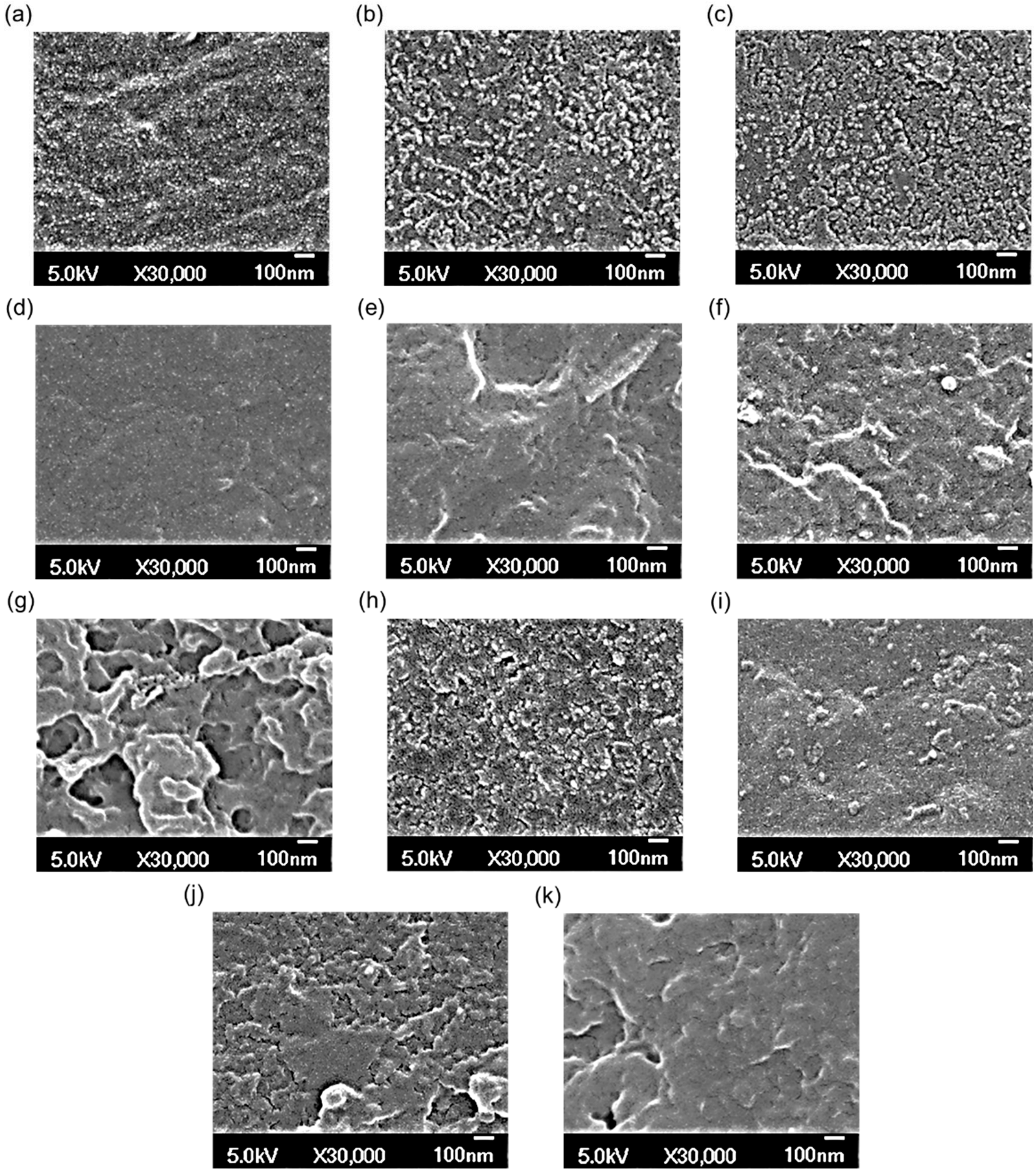

Figure S5. SEM image of the specimen surface containing potassium titanate/acrylate composites. (a) Specimen printed with resin containing 0 wt% of potassium titanate, (b) 0.5 wt% of potassium titanate, (c) 1.0 wt% of potassium titanate, (d) 1.5 wt% of potassium titanate, (e) 2.5 wt% of potassium titanate, (f) 3.0 wt% of potassium titanate, (g) 5.0 wt% of potassium titanate, (h) 0.5 wt% of potassium titanate and 10 wt% PEG, (i) 1.0 wt% of potassium titanate and 10 wt% PEG, (j) 1.5 wt% of potassium titanate and 10 wt% PEG, and (k) 2.5 wt% of potassium titanate and 10 wt% PEG.
